# Epigenetic Mechanisms in Gastric Cancer: Potential New Therapeutic Opportunities

**DOI:** 10.3390/ijms21155500

**Published:** 2020-07-31

**Authors:** Matteo Canale, Andrea Casadei-Gardini, Paola Ulivi, Maria Arechederra, Carmen Berasain, Pier-Luigi Lollini, Maite G. Fernández-Barrena, Matías A. Avila

**Affiliations:** 1 Biosciences Laboratory, Istituto Scientifico Romagnolo per lo Studio e la Cura dei Tumori (IRST) IRCCS, 47014 Meldola, Italy; matteo.canale@irst.emr.it (M.C.); paola.ulivi@irst.emr.it (P.U.); 2 Department of Oncology and Hematology, Division of Oncology, University of Modena and Reggio Emilia, 41125 Modena, Italy; 3 Program of Hepatology, Center for Applied Medical Research (CIMA), University of Navarra, 31008 Pamplona, Spain; macalderon@unav.es (M.A.); cberasain@unav.es (C.B.); magarfer@unav.es (M.G.F.-B.); 4 IdiSNA, Navarra Institute for Health Research, 31008 Pamplona, Spain; 5 National Institute for the Study of Liver and Gastrointestinal Diseases (CIBERehd, Carlos III Health Institute), 28029 Madrid, Spain; 6 Laboratory of Immunology and Biology of Metastasis, Department of Experimental, Diagnostic and Specialty Medicine (DIMES), University of Bologna, 40126 Bologna, Italy; pierluigi.lollini@unibo.it

**Keywords:** gastric cancer, epigenetic mechanisms, epigenetic therapies

## Abstract

Gastric cancer (GC) is one of the deadliest malignancies worldwide. Complex disease heterogeneity, late diagnosis, and suboptimal therapies result in the poor prognosis of patients. Besides genetic alterations and environmental factors, it has been demonstrated that alterations of the epigenetic machinery guide cancer onset and progression, representing a hallmark of gastric malignancies. Moreover, epigenetic mechanisms undergo an intricate crosstalk, and distinct epigenomic profiles can be shaped under different microenvironmental contexts. In this scenario, targeting epigenetic mechanisms could be an interesting therapeutic strategy to overcome gastric cancer heterogeneity, and the efforts conducted to date are delivering promising results. In this review, we summarize the key epigenetic events involved in gastric cancer development. We conclude with a discussion of new promising epigenetic strategies for gastric cancer treatment.

## 1. Introduction

Gastric cancer (GC) represents one of the most challenging issues for medical oncology, with 1 million people affected worldwide and patient 5-year survival rates ranging from 5 to 69%, depending on the stage of the disease at diagnosis [1,2]. Incidence and mortality rates are highly variable by region, as Eastern countries register higher morbidities. GC is influenced by several risk factors such as diet, active tobacco smoking, and *Helicobacter pylori* infections, recognized as the main risk factor for about 90% of newly diagnosed non-cardia gastric cancers [3,4]. The disease is characterized by a wide heterogeneity at the histopathological, onset location, and molecular levels, resulting in a complex scenario for patients’ clinical management and prognosis. Current treatment algorithms for GC are not able to effectively face this heterogeneity, thus creating a need for precision medicine strategies. Regarding genetic features, gastric cancers are defined by remarkable epigenetic alterations playing an active role both at the early stages of carcinogenesis and in the advanced disease. Several studies have highlighted the role of epigenetic dysregulation in GC onset and progression, in particular focusing on which driver epigenetic mechanisms could be targeted as a therapeutic approach for GC treatment [5,6]. Despite this, to date no epigenetic therapies are available for GC clinical management, and given the importance of the gastric epigenome as a main point for molecular pathogenesis and progression, effective epigenetic treatments could open a new landscape for management of the disease.

## 2. Gastric Cancer

GC is the 3rd most diagnosed and the 5th deadliest malignancy worldwide, accounting for 1 in every 12 cancer-related deaths [1]. Even though the majority of GCs are histologically classified as adenocarcinomas, GC is a heterogeneous disease that presents through different phenotypes, growth patterns, anatomic locations, and molecular characteristics, and therefore different classification systems have been proposed.

### 2.1. Anatomical, Histological, and Molecular Classification of Gastric Cancer

Gastric carcinogenesis is triggered by the interaction of different risk factors, and emerges through sequential histopathologic stages, including chronic gastritis, atrophic gastritis, intestinal metaplasia, dysplasia, and cancer [7,8]. As other luminal gastrointestinal organs, stomach cells undergo a rapid and continuous turnover, with the multipotent stem cells residing at the top of the renewal pyramid and governing organ homeostasis [9]. Hence, for their longevity and self-renewal properties, it has been suggested that gastric stem cells could represent the GC cells of origin, being ideal targets for the accumulation of genetic alterations and field cancerization, and the expansion of pro-tumorigenic mutant clones [9,10]. Interestingly, it has been highlighted that pre-cancerous lesions are characterized by a distinctive epigenetic field cancerization, mainly influenced by *H. pylori* infection [11,12].

Classification based on cancer anatomical location identifies cardia (gastroesophageal junction) and non-cardia (true gastric) tumors, which also differ in terms of incidence, regional distribution, treatment, and prognosis [13]. Cardia GCs following the Siewert classification consist of three types of cardia cancers (Siewert type I, II, or III) on the basis of the location of the epicenter of tumor with respect to gastroesophageal junction [14]. More recently, Tumor-node-metastasis (TNM) staging stystem introduced further parameters to identify gastroesophageal carcinomas, taking into account the tumor epicenter and the location where the tumor mass extends [15].

The Lauren classification, based on histological features, divides GCs into diffuse-, intestinal-, and mixed type, depending on tissue architecture and glandular patterns. Diffuse-type identifies non-cohesive and poorly differentiated tumors, with no gland formation, while intestinal-type tumors are moderate to differentiated tumors, with glandular structure not strictly related to a specific risk factor. Mixed type presents intermediate or characteristics from both previous types [16]. 

The successive WHO classification identifies five GC subtypes, mainly depending on the histological patterns of the tumor, that is, tubular, papillary, mucinous, and poorly cohesive subtypes and rare variants have been identified. Tubular carcinomas are characterized by low- to high-grade nuclear atypia with poorly differentiated cancer cells, distinguished from the papillary subtype that presents with finger-like processes of cuboidal or cylindrical cells. Mucinous carcinomas are so classified with the identification of 50% extracellular mucin, while poorly cohesive tumors have cancer cells alone or organized in small aggregates; this subtype includes signet-ring cells tumors. The mixed tumors, as their name implies, include a heterogeneous mix of the previous subtypes [17]. This classification sometimes overlaps with the one proposed by Lauren (tubular and papillary subtypes correspond to intestinal-type, poorly cohesive is associable to diffuse-type), and very often different subtypes coexist in a single tumor, although in different percentages; it is thus difficult to classify them into exclusive subtypes. 

The Cancer Genome Atlas (TCGA) program proposed the first molecular approach for GC classification. Genomic profiling of 295 primary gastric adenocarcinomas identified 9% of Epstein–Barr virus (EBV) positive tumors, 22% of microsatellite unstable, 20% of genomically stable, and 50% of chromosomally stable tumors [18]. Interestingly, these subgroups showed associations with histological subtypes and tumor locations, i.e., EBV positive tumors are mostly located in the fundus or body of the stomach, with higher prevalence in men (81%); chromosomally unstable adenocarcinomas are more frequent in the gastro-esophageal junction, whereas genomically stable tumors more often present with diffuse-type histology. Another classification based on transcriptome molecular signature identified four gastric cancer subtypes on the basis of clinically relevant features. Hypermutated tumors with microsatellite instability (MSI), characterized by intestinal subtype and major location within the antrum of the organ, showed the best prognosis and lower rates of recurrence. Microsatellite-stable (MSS) tumors were divided into mesenchymal subtype associated with worst clinical outcome and highest recurrence rates, and epithelial subtype showed intermediate prognosis. MSS epithelial tumors were further divided into MSS/TP53+ and MSS/TP53-, with a better prognosis identified in the former group [19].

### 2.2. Gastric Cancer Clinical Management 

For early GC, surgery remains the best treatment option [20,21]. Total or partial gastrectomy are the most common surgical modalities, together with a lymphadenectomy [22]. Pre-, peri- and post-operative chemotherapy approaches are highlighted to improve the outcome of patients, since these treatments prolong the 5-year overall survival (OS) of patients of 10–15% [13]. 

Survival of patients with metastatic disease is very poor, ranging from 4 to 12 months [23,24]. A large set of cytotoxic compounds are commonly used for treatment of advanced GC, such as fluoropyrimidines, platinum-based agents, taxanes, epirubicin, and irinotecan. These were initially used as monotherapies, but randomized trials and a meta-analysis showed a benefit in survival achieved by combination chemotherapy [23]. To date, the most common cytotoxic strategy is the combination of a fluoropyrimidine with a platinum-based compound [25].

On the basis of the results of the Trastuzumab for Gastric Cancer (ToGA) trial, trastuzumab plus chemotherapy has been approved as a first-line therapy for patients carrying Her-2 amplification, showing a median overall survival (OS) of 13.8 vs. 11.1 for anti-Her2 plus CT and the CT arm, respectively [26]. To date, this is the only targeted therapy approved in first-line treatment for GC management, and in recently published results from a phase 2 trial, the conjugate trastuzumab deruxtecan led to significant improvement in response rates and OS in pre-treated GC patients [27]. However, this molecular targeted therapy is available only for patients with Her2 amplification/overexpression (no more than 20% in frequency) [13]. The VEGFR-2 inhibitor ramucirumab, even though it did not confer survival benefit as a first-line treatment, was approved as a second-line treatment alone or in combination with paclitaxel, depending on the performance status of patients, on the basis of the results of the REGARD and RAINBOW trials [28,29]. Other precision medicine approaches have been attempted or are still under investigation, e.g., targeting of EGFR, VEGFR, FGFR, or the HGF receptor c-Met, but no significant improvements in OS of patients have been reached [30,31]. 

Immunotherapeutic strategies for GC are still under investigation, with some interesting emerging indicators of evidence [32]. It was reported that PD-L1 expression is related to patient prognosis and response to immune checkpoint inhibitors (ICIs), and patients with EBV positive and MSI tumors could benefit from ICI treatment, for the increased number of neo-antigens and consequent immunogenicity [33]. However, none of these biomarkers has been validated, and results from large clinical trials are needed to confirm the use of immunotherapy as a therapeutic option for gastric cancer treatment.

## 3. Epigenetics of GC

Epigenetic alterations are recognized to be both early tumor-promoting and advanced-stage events in GC [34]. Environmental and genetic factors, such as diet, age, smoking, and chronic inflammation consequent to *H. pylori* and EBV infections, are able to remodel gastric epigenetic machinery, actively paving the way for gastritis and ulcer development until metaplasia, dysplasia, and tumor development [34]. Another study analyzed the mutation status of 55 cancer-related genes, and a total of 485,512 methylation spots (482,421 in CpG sites and 3091 in non-CpG sites), finding that epigenetic aberrations could affect many cancer-related pathways [35]. Thus, there is an increasing interest about GC epigenetic events, aiming to better understand GC physiopathology and, more importantly, to find relevant targets for translational medicine. In this context, recent investigations proposed new classifications of GCs based on different epigenetic profiles rather than on somatic alterations subtyping, identifying gene methylation panels able to predict the prognosis of patients and the risk of GC metastasis [36,37,38]. In this section, we discuss the main histone and DNA epigenetic modifications characterizing GC, while the role of non-coding RNAs and their potential therapeutic interest in gastrointestinal cancers have been recently reviewed elsewhere [39].

### 3.1. DNA Methylation

Repeated CG dinucleotide sequences, often found in CpG islands (CGIs), are located in the promoter region of half of the genes, playing a central role in gene expression regulation. Methylation occurring at the 5-position of cytosines within CpG dinucleotides is a reversible process catalyzed by DNA methyltransferases (DNMTs), resulting in the formation of 5-methylcytosine (5mC) and gene expression inhibition. The methylation process is reverted by ten-eleven translocation (TET) proteins, that demethylate DNA oxidizing 5-mC to 5-hydroxymethylcytosine (5-hC), and can re-activate gene expression [40,41]. Through the TCGA molecular characterization, two subgroups of tumors with high methylation levels at multiple loci emerged, both identified as CpG island methylator phenotype (CIMP). These subgroups showed distinct methylation profiles and belong to EBV-positive tumors and the MSI subtype, referred to as gastric CIMP [18,42]. As other malignancies, GCs present global genomic DNA hypomethylation accompanied by focal hypermethylation. Generally, global hypomethylation is responsible for proto-oncogene activation and genomic instability, whereas focal hypermethylation has been implicated in turning off tumor suppressor genes.

Loss of oncosuppressor *CDH1* is a major feature of GC. Promoter hypermethylation, loss of heterozygosity (LOH), somatic mutations, and deletions affecting this gene have been related to both intestinal and diffuse GC, as well as germline mutations are considered to be the genetic cause of hereditary diffuse GC [43,44,45]. Interestingly, methylation of *CDH1* promoter has been found in 50% of hereditary diffuse GCs, and generally cooperates with genomic alterations, acting as a “second hit” to definitively silence the gene [43,46,47,48]. *CDH1* encodes for the adhesion molecule E-cadherin, and its loss triggers cancer-related pathways such as β-catenin and Wnt-, EGFR-, and mTOR-dependent signaling cascades [49,50]. *CDH1* hypermethylation is an early event in GC onset. It has been strictly related to *H. pylori* infection [51,52,53], and has also clinical significance, being able to predict worse (OS) and disease-free survival (DFS) of patients [54].

Important methylation-altered genes in GC are those involved in DNA mismatch repair (MMR) pathway. This process has a central role in maintaining the stability of the genome [55,56], and its epigenetic deregulation has been highlighted in various tumors including sporadic GC, while gene mutations affecting the main genes of the process are considered the molecular fingerprint for hereditary gastric disorders (i.e., Lynch syndrome) [57]. Methylation of promoter regions of *MLH1* and *MLH2* has been related to GC onset and progression in 108 GC specimens, and to chemoresistance to oxaliplatin [58]. Interestingly, methylation of *MLH1* predicted poor OS for advanced-stage GC patients, especially when combined with loss of oncosuppressor O(6)-methylguanine-DNA methyltransferase in two different cohorts of 135 and 68 GC patients (*MGMT*), while it was found to be a biomarker of better prognosis in resectable GC patients [59,60]. As expected, loss of *MLH1* is frequently observed in the gastric CIMP subgroup, having a strong relation with MSI tumors [18].

Several studies reported that aberrant methylation affects genes involved in cancer-related pathways able to influence the prognosis of GC patients. These include hypermethylation of *RASSF1A*, involved in cell cycle regulation, hypomethylation of *HRAS*, a component of RAS pathway [58,61,62], hypermethylation of the negative regulator of β-catenin/Wnt pathway *DKK3* [63], and hypomethylation of proto-oncogene *c-MYC* [62].

The *CDKN2A* gene encodes for p16, that inhibits CDK, resulting in cell cycle arrest, and has often been found as target for promoter methylation in GC and other gastrointestinal and solid malignancies [64,65,66]. Moreover, methylation of the *CDKN2A* promoter was also found in gastric pre-cancerous lesions in association with *H. pylori* and EBV infections, demonstrating that it could be implicated in gastric carcinogenesis [67,68,69]. For these reasons, methylation of this gene has been investigated as a biomarker of the prediction of cancer development in 207 non-neoplastic patients and, together with *CDH1*, was found to be a promising liquid biopsy biomarker for early cancer diagnosis and prognosis prediction [70,71,72,73].

Even though *RUNX3* is not frequently mutated in GC, the loss of *RUNX3* is involved in GC development [74]. The promoter region of this gene was found hypermethylated in most of the patients affected by GC (75 GC patients), with respect to cases of gastritis or non-neoplastic tissues (99 and 109, respectively) [75]. Key epigenetically deregulated genes in gastric cancer are reported in Table 1.

DNA methylation is an enzymatic reversible process catalyzed by a family of DNMTs. DNMT1 is responsible for maintaining the symmetrically methylated CGIs during DNA replication, with a role in genomic imprinting. DNMT3A and DNMT3B are able to act as de novo DNA methyl transferases, whereas DNMT2 has been identified as a tRNA methyltransferase [76,77,78,79]. DNMTs play a pivotal role in gene transcription regulation during normal development, and although expression itself does not necessarily mean increased functionality, aberrant DNMT expression has been related to carcinogenesis in almost all malignancies, including GC, as inactivation of several tumor suppressor genes occurs in a DNMT-dependent manner [80,81]. In a study by Yang et al., DNMTs were found highly expressed in GC tissue specimens (54 patients). Interestingly, DNMT1 expression correlated with cardia or body of the stomach localization of the tumor, DNMT3A expression correlated with TNM score, and their co-expression showed a correlation with lymph-node metastasis [82]. Mutze et al. found that low DNMT1 expression predicted better OS and clinical response in 127 patients treated with adjuvant therapy [83], and a comprehensive meta-analysis found that tumor tissues are characterized by high expression levels of DNMT1, with respect to normal, para-cancerous, and dysplastic tissues. Moreover, DNMT1 was found upregulated in stage III and IV patients, associated with GC risk and worse prognosis [84]. Accordingly, the TCGA molecular classification identified DNMT1 as the most upregulated among all DNMTs in each molecular subgroup [41], suggesting that it could be a common molecular driver for different pathogenesis patterns.

Other studies focused on the different DNMT gene polymorphisms, finding conflicting results about possible associations between specific a single-nucleotide polymorphism (SNP) and DNMT activity and prognosis of patients, [85,86,87,88,89,90], with the DNMT1 rs16999593 variant emerging as associated with enhanced risk of GC development in two different meta-analyses [91,92].

Multiple pathways in gastric carcinogenesis regulate DNMT expression. *H. pylori* and EBV infections result in chronic inflammation within the gastric mucosa, affecting epigenetic machinery and modulating DNMT expression through the release of oncogenic proteins such as CagA,,and inflammatory responses mediated by tumor-associated macrophages (TAMs) [12,81]. Moreover, DNMT1 expression is regulated by the tumor suppressor APC through the downstream pathway APC/β-catenin/TCF [93], and mutation or loss of APC may result in uncontrolled DNMT1 expression. Interestingly, while in colon cancers the *APC* gene is recurrently affected by somatic mutations, it has been found that *APC* promoter hypermethylation is a frequent event in GC patients, even though somatic mutations were also found in a small percentage of patients [94,95].

*H. pylori*, the only carcinogenic bacterium recognized by the WHO, triggers a series of inflammatory responses within the mucosal microenvironment, including the release of pro-inflammatory cytokines, such as IL-1, IL-6, IL-8, and TNF-α, and the activation of the NF-κB pathway [3,96,97]. Moreover, phosphorylated bacterial protein CagA is able to interact with SH2-containing phosphatase (SHP-2), inducing cytoskeleton and morphological changes via inactivation of focal adhesion kinase (FAK), disturbing the tissue architecture and leading the so-called hummingbird phenotype, which is involved in gastritis patterns and cancer development [98,99]. The activation of such pathways leads to sustained chronic inflammation within the mucosal microenvironment, and several studies reported that *H. pylori* infection is able to actively shape a pro-oncogenic epigenetic profile, especially through a wide hypermethylation of tumor suppressor genes, i.e., those of the SWI/SNF family [100].

### 3.2. Histone Modifications

Histones are a family of evolutionarily highly conserved basic proteins, which organize in octamers to wrap DNA into nucleosomal structures. Nucleosomes are characterized by histones projecting their N-terminal tails that can be post-translationally modified at single amino acid residues through different mechanisms. These include covalent modifications such as methylation, acetylation, phosphorylation, ribosylation, ubiquitination, and sumoylation [101,102] that are able to influence gene expression by changing chromatin accessibility to RNA polymerase II and transcription factors [103]. In this section, we discuss the two main histone modifications involved in gastric carcinogenesis which are also of interest for epigenetic therapeutic targeting, namely histone methylation and histone acetylation.

#### 3.2.1. Histone Methylation

Methylation of histone tails largely occurs at lysine residues, which could be mono- (me1), di- (me2), or tri-methylated (me3). This reversible epigenetic mechanism is catalyzed by histone methyltransferases (HMTs) and reverted by histone demethylases (HDMs). Histone methylation plays a dual role in gene expression regulation because, depending on the specific amino acid residue and the number of methyl groups bound, this epigenetic mechanism leads to repression or activation of gene transcription [102]. Indeed, in histone H3, methylation at lysines H3K9 and H3K27 is associated with gene silencing, whereas methylation at H3K79 is associated with transcription activation [104]. DNA and histone methylation are paired and cooperating mechanisms, with DNMTs and HMTs involved in an intense crosstalk impacting on chromatin conformation and accessibility [105]. In fact, the H3K27 methylating enzyme EZH2 is able to recruit DNMTs and, vice versa, DNMT1 and DNMT3a are able to bind the H3K9 histone methyltransferase KMT1A. Moreover, it has been shown that DNA promoter regions enriched in H3K27me3 are hypermethylated in GC cell lines such as BGC-823 and AGS [106,107]. Similar to DNA methylation, deregulation in histone modifications has been linked to gastric carcinogenesis and tumor progression. High H3K9me3 levels have been associated with T stage and gastric cancer recurrence, and it was also able to predict a worse prognosis of a cohort of 261 GC patients [108]. Aberrant histone methylation has been linked to upregulation of genes involved in cell–basement membrane and cell–cell adhesions, such as *LAMB3* and *LAMC2*, and *CLDN4* [109]. The H3K4 demethylase KDM5B has been found upregulated in GC tissues (45 paired GC tissues and adjacent non-cancerous tissues), and its ability to promote tumor growth and metastasis was demonstrated in vitro [110]. Two Jumonji C-domain demethylases, KDM3A and KDM4A, were significantly associated with TNM stage and revealed to be independent prognostic factors for OS of two different cohorts of 90 and 120 gastric cancer patients [111,112]. One of the most studied HMTs is EZH2, a Polycomb complex protein that methylates H3K27. This enzyme has been shown to interact with DNMTs and shape a carcinogenic methylation profile, and it was found upregulated in many malignancies including GC, predicting worse prognosis of patients and modulating the expression of E-cadherin in vitro [107,113,114].

In the last few years, several data reported the role of EHMT2 (G9a), the HMT targeting H3K9 and H3K27, in promoting carcinogenesis of several malignancies and in predicting depth of infiltration, lymphatic invasion, TNM staging, and prognosis of patients, including patients with GC [115,116,117]. Inhibition of G9a in GC cell lines suppressed cell growth via cell cycle arrest and autophagy. Interestingly, the authors of that study found a direct control of G9a on mTOR expression, linked to mono- but not di-methylation of H3K9, which was decreased after G9a inhibition [118]. Moreover, G9a can interact with other epigenetic molecules and be recruited to form transcriptional regulatory complexes, e.g., DNMT1/UHRF1/G9a, that coordinates maintenance DNA methylation during replication of somatic cells, or DNMT1/UHRF1/HDAC1/HDAC2/G9a, whose role is still debated [119,120]. Recently, it was demonstrated that upregulated G9a forms a functional complex with p300 and glucocorticoid receptor that induces the expression of *ITGB3*. Interestingly, G9a catalytic activity is not needed for this effect, but this complex promotes cell invasion and migration in GC cell lines, suggesting that it could be a tumor biomarker for targeted therapy [121].

#### 3.2.2. Histone Acetylation

Acetylation at lysine residues of histone tails is an epigenetic mechanism that promotes euchromatin conformation and gene expression activation. This reaction is catalyzed by a family of histone acetyltransferases (HATs), and reverted by the so-called histone deacetylases (HDACs) [101]. On the one hand, HATs are a large family of enzymes divided into three main subfamilies, each one targeting a preferential substrate—the GNAT family mainly targets H3, the MYST family mainly targets H4, whereas p300/CREB-binding protein targets both histones. Interestingly, it has been reported that acetylation can also occur on non-histone substrates, a mechanism often affecting cancer-related pathways [122,123]. On the other hand, HDACs are divided into the following four classes: class I (HDAC 1, 2, 3, 8, with mainly nuclear localization), class IIa and IIb (HDACs 4, 5, 7, and 9, and 6 and 10, respectively, with no preferential localization nucleus/cytoplasm), class III (including the sirtuins), and class IV (HDAC 11) [124]. Deregulation in HDAC expression has been linked to carcinogenesis, as HDAC aberrant expression has been found in several malignancies in association with the silencing of tumor suppressor genes [125]. In GC, global hypo-acetylation has been linked to HDACs’ class I aberrant expression [126], and reduced levels of acetylated H4 have been found in 72% of 18 GC patients, significantly correlated with T stage, tumor depth invasion, and lymph node metastasis [127]. Elevated levels of HDAC1 have been found in GC specimens compared to adjacent tissue, with a correlation with TNM stage [128]. In another study, HDAC2 levels were associated with neoadjuvant chemoresistance and higher tumor grade [129]. In both studies, HDAC1 levels were associated with worse OS of patients [128,129]. Noguchi et al. were able to correlate high levels of sirtuin1 (class III HDAC) with advanced tumor progression and worse prognosis in a large case series of patients, and they also found decreased levels of p53 expression and histone acetylation at H4K16 and H3K9 [130]. Another evidence that HDACs could prevent apoptosis in GC is provided by the capability of HDAC3 to directly downregulate *PUMA* (p53-upregulated mediator of apoptosis) gene expression in GC cell lines, with the inhibition of HDAC3 thus restoring *PUMA* expression. Moreover, the authors found elevated levels of HDAC3 in GC specimens, predicting a significant decrease in OS of patients [131]. Similar results were achieved by Feng and colleagues who found that downregulation of *PUMA* in GC specimens was correlated with decreased OS of patients, and that HDAC3 inhibition alone was able to restore *PUMA* expression and trigger p53-mediated apoptosis [132].

## 4. Current and New Epigenetic Strategies for Gastric Cancer Treatment

As epigenetic aberrations are a relevant hallmark in GC onset and development, several approaches for epigenetic treatment have been proposed (Table 2). As occurs for other solid malignancies, and in spite of numerous preclinical investigations, these therapies have not reached clinical practice yet, albeit there are some interesting emerging indicators of evidence.

To date, two classes of epigenetic drugs achieved the best results in experimental GC treatment, namely DNMT inhibitors (DNMTi) and HDAC inhibitors (HDACi). DNMTi are distinguished into nucleoside (e.g., 5-azacitidine and 5-aza-dC or decitabine (DAC)) and non-nucleoside (hydralazine) analogues, depending on their ability to integrate in the newly synthesized DNA [144]. Compounds from the former group are the only FDA-approved epigenetic monotherapies for the treatment of hematological malignancies. However, their efficacy in solid tumors remains low. This poor performance may be related to their high metabolic clearance in vivo and their instability within the acidic tumor microenvironment of solid tumors [145]. Nevertheless, more promising results in clinical trials have been achieved through combination therapies [145]. The therapeutic effect of 5-aza-dC was tested in *H. pylori*-positive gerbils, with a consistent diminution of GC incidence and a decreased overall CGI methylation. Interestingly, treatment with 5-aza-dC induced diminished levels of IL-1β and NOS2 and upregulation of TNF, a CGI-lacking gene not affected by methylation [133], suggesting that this treatment is able to reprogram the *H. pylori*-dependent oncogenic chronic inflammation. The same cancer-preventing effect was demonstrated in mice treated with carcinogen *N*-nitroso-*N*-methylurea (MNU), together with a restoration of the proto-oncogenic axis *Gdf1*-SMAD2/3, frequently found activated in GC [134].

DAC was able to inhibit matrix metalloproteinases 2 and 9 (MMP-2 and MMP-9) activity through the upregulation of their inhibitors TIMP-1 and TIMP-2 in vitro, reducing invasiveness of cells. More importantly, DAC treatment reduced the levels of pAKT, implicated in tight junction dynamics and MMP activation [135]. Another study tested the effect of DAC on 17 GC cell lines, finding an increased reduction in cell growth in the 17 CIMP cell lines [136].

The great potential of DNMTi in GC stands in overcoming resistance in chemotherapy and radiotherapy treatments. In fact, aberrant methylation patterns, especially in tumor suppressor genes involved in programmed cell death processes, have been linked to chemoresistance to 5-FU, platinum-based and irinotecan treatments, and resistance to radiation therapies [81]. In this setting, priming with 5-azacitidine prior to standard chemotherapy has been investigated in a clinical trial. Patients with resectable gastro-esophageal cancers were treated with the epigenetic agent prior to neoadjuvant epirubicin–oxaliplatin–capecitabine, achieving an overall response rate of 67%, and 25% benefited of complete response. Interestingly, the authors demonstrated hypomethylation of tumor-associated loci for all doses of 5-azacitidine, and that hypomethylation levels tended to be associated with the therapeutic response [142]. In another study conducted on five GC cell lines, epigenetic treatments were also able to increase radiosensitivity in three of them, re-establishing the expression of tumor suppressor genes involved in apoptosis [146]. In this direction, further studies are needed to better understand the interaction of epigenetic treatments and radiation therapy, given that these interesting results seem to be cell-type associated. Thus, despite the described side effects of epigenetic agents [140], the combination of these with chemo- and radiotherapy is a promising strategy and a hot topic for GC treatment, to maximize the potential of cytotoxic agents and radiation therapy. In recent years, non-nucleoside epigenetic compounds are attracting growing interest, because of their lower toxicity and the ability to bind and inhibit the catalytic domain of DNMTs, without integrating in DNA, and thus avoiding the non-specific effects of nucleoside analogues [102].

In GC, most of the preclinical evidences of epigenetic treatment have been provided through HDAC inhibition. HDACi compounds are biochemically divided into the following four classes: short-chain fatty acids, hydroxamates, cyclic tetrapeptides, and benzamides [124]. 

Like DNMT inhibition, HDACi are able to synergize with chemotherapeutic agents [129,141,147] and radiation therapy [6]. For this reason, the ability of HDACi to act as priming drugs for chemotherapy agents was investigated. A recent study showed that treatment of the GC cell line AGS with different HDACi prior to chemotherapy agents resulted in a better binding of chemotherapies to chromatin, with lower doses needed to achieve maximum efficacy when the drugs were administered in combination [126]. However, data from a phase II clinical trial combining HDACi (Vorinostat) with capecitabine–cisplatin in advanced GC patients showed an objective response rate of 42%, not appearing to improve the clinical outcome of patients, and with a considerable rate of grade 3–4 adverse events [143].

Another preclinical study proved that HDACi treatment re-established the expression of tumor suppressor genes *PER1* and *PER2*, mainly known as circadian regulators, that are involved in cell cycle arrest, apoptosis, and loss of clonogenic activity [137]. Valproic acid, largely used as a anticonvulsant drug, has been recently studied for its HDACi ability, and it was demonstrated to target HDAC1/2 and the HDAC1/PTEN/Akt axis in GC cell lines, inhibiting cell growth and triggering apoptosis [138].

A role for HDAC9 as a targetable biomarker has been recently proposed by Xiong et al., highlighting its aberrant expression and a correlation with patients’ survival. Interestingly, the authors found that pharmacological targeting of HDAC9 inhibits cell survival and induces cell cycle arrest with consequent apoptosis, synergizing with the effects of cisplatin [141]. Similarly, Dong et al. uncovered the anticancer effects of a specific HDAC6i, able to induce cell cycle arrest and apoptosis. Moreover, the authors observed a decrease in neo-angiogenetic biomarkers and a loss in mitochondrial membrane potential [139]. Since not all epigenetic targets are equally expressed in GC, these are nice examples that targeting a specific epigenetic effector could be a good strategy for better tailoring precision medicine and possibly diminishing the side effects observed with pan-HDACi [143]. In fact, the therapeutic mechanism of action of HDACi is not fully understood, and HDACs have a wide range of targets, not only increasing histone acetylation, but also through a plethora of antitumoral mechanisms, including direct cytotoxic effects [6]. Interestingly, in a preclinical model of hepatocellular carcinoma, treatment with an HDAC pan-inhibitor resulted in the downregulation of DNMT expression and activity [148].

Considering the side effects of a pan-inhibition, and that epigenetic mechanisms often cooperate with each other to shape an aberrant profile, a targeted dual inhibition of epigenetic mechanisms could be an attractive strategy to test in GC models. A dual targeting of two HMTs, EZH2 and G9a, was performed in preclinical models of breast cancer. The authors of that study demonstrated a global restoration of gene expression, and inhibition of cell growth. Moreover, they found that the dual inhibition achieved the re-expression of a subset of genes that would not be re-expressed with a single agent [149]. Nevertheless, further studies are needed to assess the toxicity profile of such treatment. In GC cells, depletion of HMT G9a effectively reduced levels of histone methylation and triggered apoptosis [117], and, as in the case of other epigenetic treatments, increased the chemosensitivity of cells to 5-FU [150]. Moreover, G9a can be recruited by other epigenetic inhibitors to exert autophagy-mediated apoptosis [151], and can form transcriptional regulator complexes with DNMTs, maintaining an active crosstalk with these molecules [120,152]. Moreover, Wozniak et al. demonstrated, in breast cancer cells, that G9a is regulated by 5-aza-dC through a dose-dependent post-transcriptional mechanism, and the addition of siRNA blocking both G9a and DNMT1 resulted in increased expression of tumor suppressor genes, suggesting that multiple layers of epigenetic deregulation cooperate in a single cellular context [153]. Dual inhibition of G9a and DNMT1 has been successfully attempted in preclinical models of hepatocellular carcinoma. The effective antitumoral activity in vitro and in vivo of the tested compound was demonstrated, with a synergistic effect with chemotherapy and other epigenetic drugs. Interestingly, dual targeting of G9a and DNMT1 reprogrammed the metabolic adaptation to hypoxia of cancer cells, with a diminished glucose intake and a general diminished expression of glycolytic enzymes [154]. Similar to other malignancies, GC is addicted to glucose consumption, aerobic glycolysis, and the establishment of an acidic microenvironment through accumulation of lactic acid, resulting in a growth advantage for cancer cells via adaptation to hypoxia [155]. In this scenario, it could be useful to clarify how epigenetic targeting could remodel metabolic activity of cancer cells, forcing them to a less advantaged condition and a less aggressive metabolic behavior.

In the era of immuno-oncology, GC has revealed to be a tumor with weak immunogenicity, and despite encouraging results, the response rates in clinical trials with immune checkpoint inhibitors remain limited [32,156]. In this setting, emerging data on immunotherapy for GC highlight that expression of immune biomarkers is epigenetically regulated, and that epigenetic mechanisms are able to predict clinical response to immune checkpoint inhibitors [157,158,159,160]. Moreover, the aberrant epigenome of GC was revealed to contribute to cancer immunoediting and to immune escape of cancer cells [161]. Conversely, activated immune cells were able to induce the downregulation of tumor suppressor genes through DNMT1 recruitment and activation [162]. Aberrant epigenome of cancer cells and an exhausted T-cell tumor infiltrate are features deeply characterizing GC, and represent a possible walkable way for new treatment strategies. Moreover, in CIMP gastrointestinal malignancies and other non-gastric tumors, epigenetic strategies targeting the cancer epigenome could increase cancer immunogenicity likely to respond to immunotherapeutic agents by reprogramming the tumor immune microenvironment. Moreover, a recent study in GC demonstrated that unresponsiveness to anti-PD-1 antibodies could be the result of epigenetic silencing of PD-L1 [163,164,165,166]. Hence, in the near future, an attractive approach could be a strategy to test the combination of epigenetic and immunotherapeutic agents in GC models.

## 5. Conclusions

**Figure 1:**
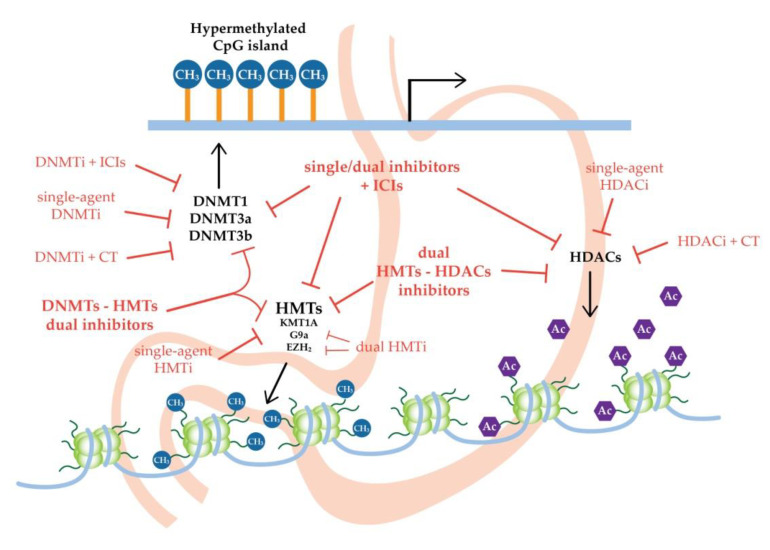
Promising new epigenetic strategies for gastric cancer treatment. CT: chemotherapy; DNMTs: DNA methyltransferases; DNMTi: DNA methyltransferase inhibitor; HDACs: histone deacetylases; HDACi: histone deacetylase inhibitor; HMTs: histone methyltransferases; HMTi: histone methyltransferase inhibitor; ICIs: immune-checkpoint inhibitors.

GC is one of the major causes of cancer-related deaths worldwide. Epigenetic mechanisms stand at the base of carcinogenesis and progression of the disease and, together with risk factors and genetic alterations, they are able to guide gastric malignancy development. However, altered epigenomic profiles could represent the cancer susceptibility for novel therapeutic strategies for gastric cancer (Figure 1). In fact, targeting epigenetic mechanisms could overcome cancer heterogeneity and reprogram the cancer homeostasis likely to respond to cytotoxic agents or immune checkpoint inhibitors. In this way, it could be possible to combine several epigenetic strategies, or use them with standard chemotherapies or immunotherapies.

## Figures and Tables

**Table 1:** Key epigenetically dysregulated genes in gastric cancer.

Target	Role	Ref.
*CDH1*	Cell–cell adhesion	[47]
*MLH1*, *MLH2*	DNA repair	[58]
*MGMT*	DNA repair	[59]
*DKK3*	Wnt signaling pathway regulation	[63]
*RADSSF1A*	Cell cycle regulation	[61]
*HRAS*	Component of RAS pathway	[62]
*c-MYC*	Transcription factor	[62]
*CDKN2A*	Cell cycle regulation	[64]
*RUNX3*	Transcription factor	[73]
*VEGF-c*	Neo-angiogenesis related	[76]
*GATA 4/5*	Gastrointestinal cell differentiation	[76]

**Table 2:** Examples of preclinical and clinical evidences of epigenetic strategies for gastric cancer treatment.

Treatment Strategy	Epigenetic Target	Drug	Result	Model or Clinical Study Phase	Ref.
Single-agent	DNMTs	5-azacitidine	Decreased GC incidence and decreased global hypermethylation in vivo	Mongolian gerbils	[133]
	DNMTs	5-azacitidine	Restoration of *Gdf2*-SMAD2/3 axis	MNU-treated mice	[134]
	DNMTs	DAC	Reduction of invasiveness of GC cells	GC cell lines	[135]
	DNMTs	DAC	Reduced cell growth in CIMP-positive cell lines	GC cell lines	[136]
	HDACs	TSA	Re-establishment of tumor suppressor gene expression	GC cell lines	[137]
	HDACs	VA	Inhibition of cell growth and apoptosis trigger	In vitro and in vivo models	[138]
	HDAC6	TC24	Cell cycle arrest and apoptosis, loss of mitochondrial membrane potential	GC cell lines	[139]
Combination therapy, epigenetic priming	HDACs	VPA, TSA, SAHA, chemotherapy	Increase of DNA binding of cytotoxic agents and higher cytotoxic potential	GC cell lines	[126]
	HMT G9a	G9a siRNA + 5-FU	Apoptosis trigger, synergism with 5-FU	GC cell lines	[140]
	HDAC9	HDAC9 siRNA + cisplatin	Cell cycle arrest and apoptosis, synergism with cisplatin	In vitro and in vivo models	[141]
	DNMTs	5-azacitidine prior to neoadjuvant chemotherapy	67% overall response rate, 25% complete response	Phase I (NCT01386346)	[142]
	HDACs	SAHA + capecitabine, cisplatin	42% objective response rate, increased adverse events	Phase II (NCT01045538)	[143]

Abbreviations: 5-FU: 5-fluorouracil; DAC: decitabine; DMNT: DNA methyltransferase; GC: gastric cancer; HDAC: histone deacetylase; HMT: histone methyltransferase; MNU: *N*-nitroso-*N*-methylurea; SAHA: suberoylanilide hydroxamic acid; TSA: trichostatin A; VA: valproic acid.
